# Quantitative risk assessment for human *Taenia solium* taeniasis/cysticercosis exposure through consumption of pork in Mpwapwa District of Dodoma Region, Tanzania

**DOI:** 10.14202/vetworld.2023.895-911

**Published:** 2023-05-07

**Authors:** Misheck A. Mulilo, Kabemba E. Mwape, Ethel M’kandawire, Ernatus M. Mkupasi

**Affiliations:** 1 Livestock Training Agency, Department of Training, Research and Consultancy, Mpwapwa, Dodoma, Tanzania; 2 Department of Clinical Studies, The University of Zambia, School of Veterinary Medicine, Lusaka, Zambia; 3 Department of Disease Control, The University of Zambia, School of Veterinary Medicine, Lusaka, Zambia; 4 Department of Veterinary Medicine and Public Health, Sokoine University of Agriculture, College of Veterinary Medicine and Biomedical Sciences, Morogoro, Tanzania

**Keywords:** cysticercosis, Mpwapwa, pork meal, quantitative risk assessment, *Taenia solium*, taeniasis

## Abstract

**Background and Aim:**

Pig farming is a livelihood activity undertaken by many rural communities in Tanzania. However, pigs in rural communities become infected with *Taenia solium*, a zoonotic parasite leading to porcine cysticercosis (PCC). Thus, routine meat inspection is fundamental in ensuring that the meat consumed is PCC-free. However, routine meat inspection is constrained by low sensitivity as a diagnostic test. Regardless of its low sensitivity, at the local level, no substitute tool would potentially lead to meat being risk-free for human infection. This study aimed at quantifying the risk of humans getting exposed to T. solium taeniasis through the consumption of pork approved safe for human consumption by employing a quantitative risk assessment (QRA) model.

**Materials and Methods:**

A cross-sectional study employing a quantitative risk assessment technique was conducted to quantify the risk of human infection in Mpwapwa District through exposure to *T. solium* infection through consumption of officially inspected pork. The input parameters in this study were simulated in @risk software to obtain the risk of exposure and the risk factors for exposure to *T. solium* taeniasis.

**Results:**

The risk of one getting exposed to *T. solium* taeniasis/cysticercosis (TSTC) through consumption of pork approved for human consumption was found to be 0.018 (95% confidence interval [CI] = 0.00–0.0250). Likewise, the probability that a cyst is localized in a pork portion was found to be the most influencing input risk factor of getting exposed to TSTC. Furthermore, the probability of developing *T. solium* taeniasis was estimated to be 0.73605 (95% CI = 0–0.950) when infected undercooked pork portion is consumed and 0.99652 (95% CI = 0.98161–0.99908) from consuming raw pork portion. Likewise, about 47 (95% CI = 42–52) people who consumed undercooked pork and 26 (95% CI = 22–30) who consumed raw pork would get infected in Mpwapwa District per year.

**Conclusion:**

The results from this study are anticipated to create public awareness of the problem and promote the use of one-health approach in the control and prevention of the consumption of infected pork.

## Introduction

Pig farming is an activity undertaken by many rural communities to sustain their social needs and wants [1]. However, due to the traditional husbandry practices in rural areas, pigs acquire porcine cysticercosis (PCC) through the ingestion of viable *Taenia solium* eggs in human feces or contaminated feed and water [2, 3]. The endemicity of PCC in rural areas is linked to keeping pigs under a free-range system, poor cooking of pork meat, poor sanitation and hygienic practices, open defecation, and poor meat inspection services [4, 5]. These risk factors maintain the life cycle of *T. solium* between humans and pigs. Humans, as definitive hosts, harbor the adult *T. solium* in the gastrointestinal tract. These worms undergo apolysis to shade gravid proglottids containing embryonated eggs through human feces [6]. When such excretes are openly disposed of, they become ingested by scavenging pigs and ultimately, the eggs hatch to a larval stage called *Cysticercus cellulosae*. The later disseminates into the muscle of pigs causing PCC.

In Tanzania, this disease was first detected in pigs that were exported to Kenya in the 1980s [7]. The negative impact of this disease cannot be deserted in humans due to taeniasis and neurocysticercosis (NCC) and in pigs due to economic losses. Many studies on the prevalence and burden of the disease have been carried out in Tanzania. However, most of these studies are based on areas where pigs are kept the most. Elsewhere, the disease remains underreported. The overall prevalence of PCC ranges between 6%–17.4%, 0%–18.2%, and 1.5%–33.3% by lingual palpation, post-mortem examination, and Ag-enzyme-linked immunosorbent assay (ELISA), respectively [8].

Humans can be infected with both larval stage and adult worm after ingesting the tapeworm eggs or consumption of infected pork that is not properly cooked, respectively [9, 10]. The result of such infestations in humans is *T. solium* taeniasis/cysticercosis (TSTC). Cysticercosis in humans occurs when the larvae lodge in the muscles, skin, eyes, or central nervous system [9]. The latter may result in NCC, which is among the causes of preventable epileptic seizures [11]. Epileptic seizures affect more than 50 million people worldwide, of which more than 80% of affected people live in rural areas of developing countries, especially in Latin America, South and South-East Asia, and sub-Saharan Africa [10, 12, 13]. In Tanzania, the overall prevalence of taeniasis ranges from 2.3% to 5.2% based on copro-antigen ELISA and that of human cysticercosis is 16.7% [8]. In addition, the incident cases and deaths of NCC-associated epilepsy range from 5666 to 36,227 and affect 37–612 people per year. Besides these ill health effects associated with TSTC, the monetary and societal cost of TSTC is estimated to be 5 million United States Dollars for the treatment of NCC-associated epilepsy [14].

Pig production has been increasing at a fast rate in recent years as a result of an increase in pork demand in urban areas [15]. This means that the infected pigs raised in rural areas are likely to be transported and consumed in urban areas if control efforts are not taken into consideration. Routine meat inspection and appropriate processing or cooking of pork are fundamental in preventing human infection with *T. solium* [16]. For this reason, routine meat inspection is incorporated into the food chain as a method to ascertain the safety of meat for human consumption. This method not only removes unfit meat from the food chain but also prevents the risk of exposure of the public to the infected meat. Concerning PCC, meat inspection provides a 100% specific and double advantage of being able to detect cysticercosis in live animals (pigs) through tongue palpation during an antemortem inspection and detects PCC in muscles of pig carcasses during routine post-mortem meat inspection [17]. To strengthen this protocol, different countries have laid down regulations and legislation on how it has to be carried out. The Food and Agriculture Organization (FAO), being an overseer of food safety and security worldwide, provides the procedures for conducting routine meat inspections in developing countries [18]. This has also been adopted in Tanzania whereby the Food (Control of Quality) (Slaughterhouses, Slaughtering, and Inspection of Meat) Regulation, in 1993, mandates routine meat inspection and provides the procedure and judgment patterning to *T. solium* PCC [19]. This regulation also declares that selling unofficially inspected pork intended for human consumption is illegal.

Despite the high specificity of routine meat inspection for PCC, the method is constrained by low sensitivity as it can only detect a heavy burden of *C. cellulosae* in pork [20]. This then allows lightly infected carcasses to be passed on for human consumption, thereby increasing the probability of exposing the public to *T. solium* taeniasis [21]. For instance, in Zambia, more than 60% of infected pigs were not detected during routine meat inspection and hence entered into the food chain [22]. Similarly, about 70% of infected pigs entered the food chain in Kenya and 30% of the exported pork was not detected by a routine meat inspection at the abattoir [23]. Further, 76.3% of infected pigs were not detected in South Africa [17]; similar findings have also been reported [24] in India.

This study aimed at quantifying the risk of humans getting exposed to *T. solium* taeniasis through the consumption of pork approved safe for human consumption by employing a quantitative risk assessment (QRA) model. This model uses a farm-to-fork approach to scrutinize the chain of events that affect the viability and number of viable cysts to which pork consumers are exposed through the consumption of pork officially passed for human consumption.

## Materials and Methods

### Ethical approval

The study was approved by the Tanzania Livestock Research Institute (TALIRI) with approval number TLRI/RCC.22/001. However, before undertaking the data collection, permission was sought from the local authority.

### Study period and location

**Figure-1:**
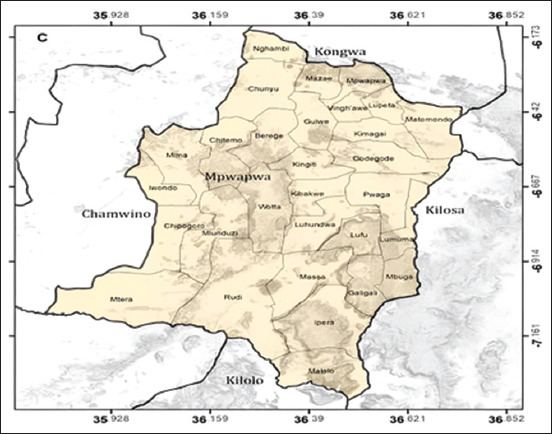
Map showing details (wards) of Mpwapwa districts and borders [25].

The study was conducted from March 2022 to July 2022 in Mpwapwa District; one of the seven districts of Dodoma region, the Capital city of Tanzania. This district is located 120 km Southwest, of Dodoma city headquarters. The district is bordered by four districts two of which are within the same region, while the other two are located in Morogoro and Iringa regions, the district borders Kilosa District in Morogoro region on the eastern and Kilolo District in Iringa region on the south. The district also borders Chamwino and Kongwa Districts on the west and the northern parts, respectively, in Dodoma region (Figure-1). Mpwapwa District lies between latitude 6°00’ and 7°30’ south of the Equator and between 35°45’ and 37°00’ east of Greenwich. It is found at 915–1200 m above sea level with a total surface area of 7379 sq. km and annual rainfall varying from 900 mm to 1200 mm per annum [25].

The district was selected due to the high number of pigs kept and the presence of risk factors for the spread and acquisition of diseases from pig to man. Mpwapwa District is the second in the number of pig production in Dodoma region keeping about 37,015 pigs. It is one of the districts which have been supplying pigs to Dar-es-Salaam; the commercial city of Tanzania [13, 15]. However, some wards in the district have no veterinary experts to inspect meat; some areas were observed to have pigs left to scavenge.

### Study design

**Figure-2:**
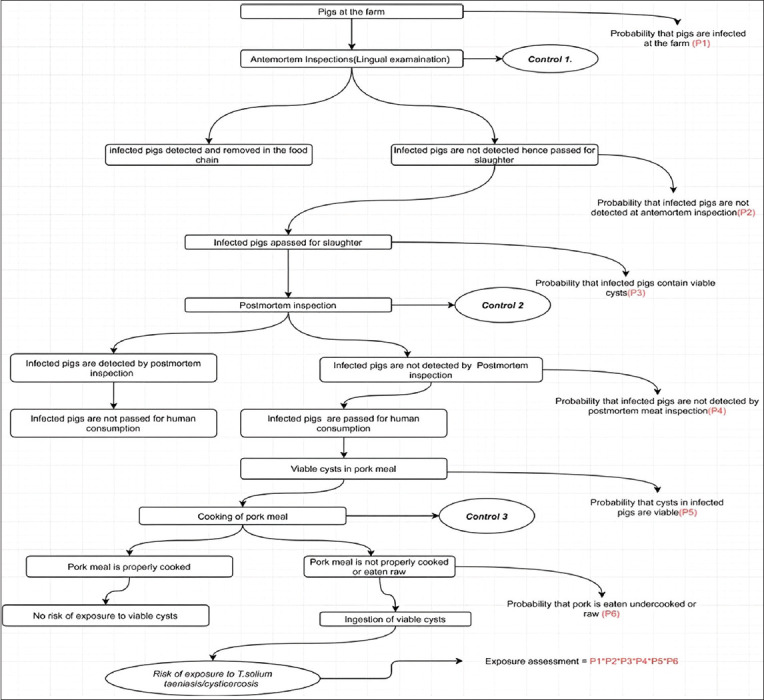
The conceptual model pathways right from the farm to consumption in the kitchen.

This was a cross-sectional study utilizing both primary and secondary data. This study employed a quantitative microbial risk assessment technique under the Codex framework to quantify the risk of a pork consumer getting exposed to TSTC through pork consumption in the study areas. A QRA model was developed from the farm to the exposure of humans to viable cysts in pork meal portions at the point of consumption following the described conceptual model pathways (Figure-2). To fill the information gap in the risk assessment, a primary study through a questionnaire survey was conducted and the generated data were used to estimate model input parameters. The primary data included the number of people who consume raw and undercooked pork, the quantity of pork consumed per person per meal, and pork preparation preferences among others.

### Sample size estimation for the questionnaire survey

Cochran’s formula for calculating sample size when the population is infinite was used to calculate the sample size [26]:


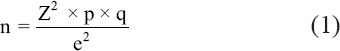


Where: n = required sample size, z is the selected critical value of desired confidence level (95% confidence level), p is the estimated proportion (prevalence of PCC in Mpwapwa District), q = 1-p and e is the desired level of precision (marginal error).

Therefore, z at 95% confidence interval (CI) is 1.96%, e = 0.08, p = 0.5 and q = 0.5:


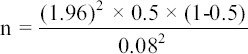



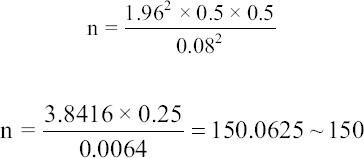


Hence, 150 people were sampled to obtain information on consumption patterns and pork preparation preferences.

### Sampling technique

A simple random sampling was used to obtain representative wards and villages from each selected division. Representative wards and villages were randomly selected by an assistant who was blinded. From each representative division, that is, Mpwapwa and Kibakwe, two wards were randomly selected and from each selected ward, two representative villages were selected amounting to eight villages from where representative respondents were obtained. The two wards selected from Mpwapwa Division included Mpwapwa and Matomondo and from Kibakwe Division included Pwaga and Kibakwe wards. Then from Mpwapwa Ward, Ilolo and Igovu villages were selected and from Matomondo Ward Mbori and Tambi villages were selected,. Moreover, from Kibakwe Ward, Kibakwe and Chamtumile villages were included and from Pwaga Ward Pwaga and Masala villages were included in the study. From these eight villages, 25, 26, 18, 19, 20, 21,11, and 10 respondents were randomly selected.

These numbers of representatives in each ward (NW) were obtained based on the proportion of the total population in each division (PD). The number of respondents from the representative village (NVP) was selected based on their proportion of the population from each respective ward as described in the formula below.


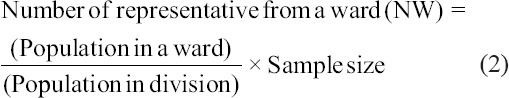


The population in Mpwapwa and Kibakwe Divisions are 112,822 and 79,336, respectively [27]. Therefore, the number of representatives from Mpwapwa Division (NWP) was deduced from the formula below:


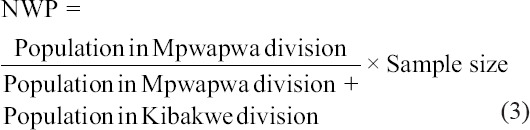



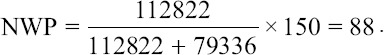


Therefore, 88 people were selected from Mpwapwa Division.

The number of representatives from Kibakwe Division (NWK) was deduced as:


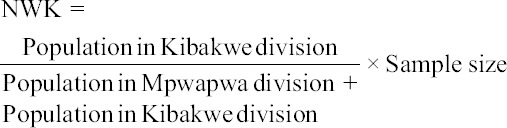



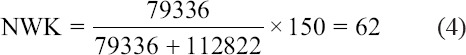


Therefore, 62 representatives were selected from Kibakwe Division.

The respondents to be interviewed from each representative village were selected based on the proportion of the population between the selected wards in the same division.


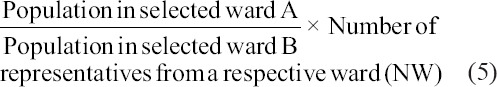


Therefore, the number of representatives from Matomondo and Mpwapwa town wards was calculated as follows, given that the population of Matomondo ward is 9792 people and 23,190, respectively.


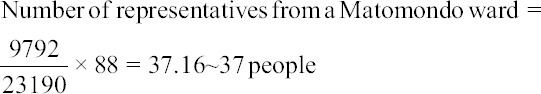


While representatives from Mpwapwa town ward will be 88–37 = 51. Following these calculations, the numbers of interview respondents (interviewees) are shown in Table-1.

**Table-1:** The number of respondents interviewed from different villages of Mpwapwa district for pork preparation preferences and consumption patterns in Mpwapwa district.

Division	Mpwapwa				Kibakwe			
Interviewees	88				62			

**Wards**	**Mpwapwa**		**Matomondo**		**Kibakwe**		**Pwaga**	

Interviewees	51		37		41		21	

**Villages**	**Ilolo**	**Igovu**	**Mbori**	**Tambi**	**Kibakwe**	**Chamtumile**	**Pwaga**	**Masala**

Interviewees	25	26	18	19	20	21	11	10

### The QRA process

The QRA was prepared following the guidelines of Miller *et al*. [28].

- State the question and the scope of the risk assessment
- Identify the hazard of interest
- Develop a scenario tree that outlines the pathway of expected events and all the failures which could occur, culminating in the occurrence of the identified hazard
- Label the scenario tree and assign units
- Gather and document evidence (Data collection and management)
- Assign values to the branches of the scenario tree
- Perform the calculations to summarize the likelihood of the hazard occurring
- Consider risk management options.

#### Risk question

The risk question was “What is the risk of one getting TSTC through the consumption of routinely inspected pork in Mpwapwa District?”

#### Scope of the risk assessment

This risk assessment was limited to estimating the risk of pork consumers getting exposed to TSTC through the consumption of pork passed for consumption in Mpwapwa District, assessing the sensitivity of routine meat inspection protocols, and estimating the number of people who might get infected through the consumption of pork per year.

#### Hazard identification

The hazard in this study was identified to be *T. solium*. This parasite is commonly known as a pork tapeworm as pigs are the significant intermediate host. The larva of this parasite, called *C. cellulosae*, in its development stage lodges in the muscles of pigs which when ultimately consumed by pork eaters without proper cooking, gets its way to the intestines of the human host as the only final host [29, 30]. With the help of its suckers and hooks, it attaches firmly to the intestinal wall where it grows to the length of 2–4 m [3, 29–32].

#### Scenario trees

Scenario tree for exposure assessment

**Figure-3:**
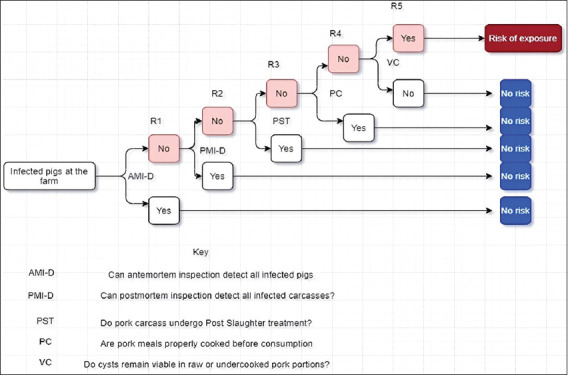
Risk pathways (R1, R2, R3, R4, R5) for exposure assessment to *T. solium* taeniasis through consumption of pork.

The pathways of *C. cellulosae* by which pork consumers become infected through the consumption of pork are the factors of the presence of infected pigs at the farm, the failure of both antemortem and post-mortem protocols to detect all infected pigs and carcasses, respectively, and the absence of post-slaughter treatment (freezing) of pork before cooking and improper cooking of pork or eating raw pork (Figure-3).

Scenario tree for hazard characterization

**Figure-4:**
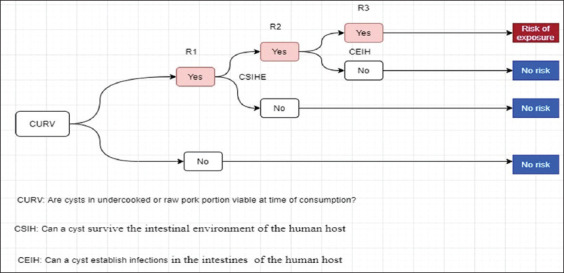
Risk pathways (R1, R2, R3) for hazard characterization part of quantitative risk assessment for the exposure to *T. solium*.

This employed input parameters that lead to the establishment of infection in the human host. The establishment of infection in the human host depends on whether the cysts are viable in undercooked or raw pork portion at consumption, the ability of cysts to survive the intestinal environment of the human hosts, and the ability of cysts to establish the infection in the human host (Figure-4).

Scenario tree for risk characterization

**Figure-5:**
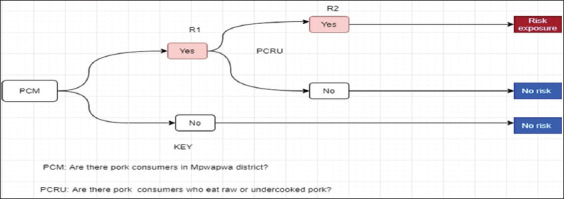
Risk pathways (R1, R2) for risk characterization assessment to exposure to *T. solium* taeniasis-cysticercosis through consumption of pork.

Exposure to *T. solium*/taeniasis through consumption in Mpwapwa is the factor of the presence of pork consumers in Mpwapwa District and whether these consumers eat undercooked or raw pork meal portions (Figure-5).

### Data collection and data management

#### Sources of data

This study, as the nature of risk analysis studies involved much information search from peer-reviewed scientific papers and journal articles as well as unpublished information (grey data) from public offices as secondary data. Primary data were collected through a questionnaire survey.

Secondary data

These data included both published peer-reviewed articles and non-published ones from public offices. The literature review was guided by the research question and the conceptual model pathways above. Peer-reviewed articles were obtained from electronic databases, mostly from Google (https://www.google.com), Google Scholar **(**https://scholar.google.com, and PubMed **(**https://pubmed.ncbi.nlm.nih.gov**).** Other types of information were collected from specific websites such as World Health Organization (https://www.who.int), OIE (https://www.oie.int), Centers for Disease Control and Prevention (https://www.cdc.gov), FAO (http://www.fao.org) and the repository database of UNZA library (http://dspace.unza.zm), Sokoine University of Agriculture Institutional Repository (http://www.suaire.sua.ac.tz), Dodoma regional website (http://www.dodoma.go.tz), and Mpwapwa District website (http://mpwapwadc.go.tz). However, information on the post-mortem records of pigs was obtained from the Mpwapwa District Veterinary Office.

Primary data

After extensive literature reading, it was found that some information on pork consumption patterns and preparation preferences in Mpwapwa District was missing. Therefore, to fill this information gap, a questionnaire survey was conducted on pork consumers in the study area. One hundred and fifty pork consumers were then interviewed from eight villages which were randomly selected from Mpwapwa and Kibakwe Divisions.

Respondents were asked about their willingness to participate in the study by signing in the consent form. The questions were prepared and administered using an Epicollect 5 Data Collection tool which is a free data collection application in the Google play store. These questions were prepared in English but translated into Kiswahili language during the face-to-face interview as most respondents were able to understand Kiswahili.

#### Data management

Data were entered into the Palisade @risk® software version 8.2.00172.0 (Palisade Company, LLC) Excel sheet containing three attributes, the parameter number, parameter description, source of the data, and their probability/proportion or the number of parameters required. The obtained information was entered into a specific row and column with their minimum, most likely and maximum values of probability or proportion with a respective probability distribution for continuous and discrete data. Risk Pert and Risk Uniform were the only function distributions used for continuous variables, while risk poisson, risk binomial, and risk in uniform were used for discrete variables. Information from the questionnaire surveys was entered into IBM statistical package for the social sciences (SPSS) statistics version 21 (IBM Corp., NY, USA) and coded for descriptive statistical analysis. The obtained secondary data were both used to formulate a model parameter input description and risk assessment model.

### Model parameter input description

Input parameters used in this study followed all activities that are used in the pork food chain right from the farm to fork, leading to exposure of TSTC to consumers at consumption.

#### Input parameters for the exposure assessment

These calculations involved inputs associated with the pork chain from farm to fork. The input parameters used for this assessment were the number of pigs slaughtered in Mpwapwa District per year, the probability that the pig is infected at the farm, the number of infected animals sent for slaughter, and the probability that an infected pig is missed by lingual inspection. Other parameters were the number of infected pigs not detected by lingual palpation, the probability that infected pigs are not detected at post-mortem inspection, the number of infected pigs not detected at post-mortem meat inspection, as well as the probability that the infected pork contains viable cysts, the number of infected carcasses containing viable cysts and the meals potions per pig/person. Besides these, exposure assessment includes other parameters such as the infective meal portions passed for consumption/year, the number of cysts per carcass, the probability that cysts in an infected carcass are viable, the number of viable cysts in a carcass, the probability a cyst is localized itself in a pork portion, as well as the number of cysts distributed in the meal pork portions. A simulation was run at 100,000 alterations until all bars converged to provide the number of viable cysts in 100 g pork meal portions just at consumption. This was then followed by estimating the likelihood of pork consumers getting exposed to these parasites in question.

#### Input parameters for hazard characterization

This involved establishing the dose-response relationship in the pork portion, considering the number of viable cysts consumed and the nature of pork portions (undercooked and raw pork portions). For this assessment, the input parameters used were the number of viable cysts ingested in undercooked and raw pork portions, respectively, the number of viable cysts that can establish infection in humans when undercooked and raw pork portions are consumed and the ability of cysts to survive the intestinal environment. However, due to a lack of human data on the dose-response relationship of TSTC, considering host susceptibility in man was not done. There is no established dose-response relationship of *T. solium* taeniasis [33]. The conduct of such studies is partly associated with ethical reasons [34]. To conduct a hazard characterization, the exponential single-hit theory model for establishing a dose-response relationship was adopted in this study. This model is useful in cases when a single hazard (single microorganism) can induce infection in the human host [35].


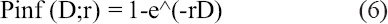


Where Pinf is the probability of infection, D means the number of ingested doses of cysts, and r means the ability of the cysts to survive in the intestines of the host.

To approximate the ability of a viable cyst to survive and establish itself in the intestine, the following assumptions were taken into consideration. First, the number of ingested viable cysts (D) to establish taeniasis was considered to be three. This was adopted from a study by Yoshino [36], who ingested three viable cysts and other volunteers ingested five viable cysts to have a constant source of *T. solium* eggs in his study. Second, the number of cysts that will succeed in growing to an adult and which will result in infection (r) was considered to be one; however, a study by Yoshino [36] recovered 2–5 adult *T. solium* from 3 to 5 ingested viable cysts with an infection rate of 60%–100%. On the other hand, Ito *et al*. [34] report this infection rate as high because cysts were not subjected to mastication as some cysts die in the process of chewing while others fail to attach following the human host immune-parasite defensive system [37]. Therefore, in this study, these factors were taken into consideration for calculating the ability of the cysts to survive in the intestines of the human host (r). Therefore, the ability of cysts to establish themselves was taken as a ratio n/c where n is the number of cysts which develops to adult (1) and c is the maximum number of viable cysts required to establish the infection (3) = n/c = 1/3 = 0.3333. Third, due to the lethal effect of cooking/heat during preparation therefore, even if the cysts are viable but their integrity could be reduced. Therefore, this study assumed that the ability of cysts to survive in the intestines given pork portion is undercooked, which is reduced to half compared to that in raw pork portions.


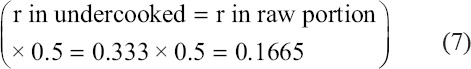


#### Input parameters for risk characterization

Monte Carlo simulation was done to model the risk of one getting exposed to TSTC through the consumption of pork approved for human consumption. The number of people getting exposed to TSTC per year and the number of people who get infected per year through the consumption of pork passed for consumption in the Mpwapwa District were also estimated. In this study, risk characterization employed the following input parameters; the population t, the number of households, and the number of households consuming pork in Mpwapwa District. Other input parameters were the number of people consuming pork, the proportion of people who consume undercooked pork/year, the number of people who consume undercooked/year, and the proportion of people who consume raw pork/year in Mpwapwa district.

### Determination of the risk factors for exposure of humans to TSTC

A sensitivity analysis was conducted to determine the risk factors for exposure of humans to TSTC. This was done by plotting the Tornado graphs which consist of a series of bars, the longer bars at the top representing the most contributing factors to the risk output [38]. To attain this, the change in output statistics and the spearman rank correlation coefficients Tornado graphs built in Palisade @risk® software version 8.2.00172.0 (Palisade Company, LLC, Ithaca, NY, USA) was used in this analysis. While the change in output statistic ranks the input based on its effects on the output mean, the Spearman rank-order correlation coefficients measure the strength of the association (relationship) between the input variable and the output by calculating their correlation coefficient (ρ) from the rank of input variables. This strength of the association varies between −1 and +1, whereas 0 indicates no association and the signs show the direction of the association. Furthermore, ±0.1–±0.3 indicates weak association, ±0.4–±0.6 indicated moderate association, ±0.7–±0.9 indicated strong association, and ±1 indicated perfect association [39].

### Risk assessment model

This was described using information that could lead to answering the risk question. Seven risks were considered to be significant; these are the probability that the pig is infected at the farm, the probability that an infected pig is missed by lingual inspection, and the probability that infected pigs are not detected at post-mortem inspection. Besides these, there is the probability that the infected pork contains viable cysts, the probability that cysts in an infected carcass are viable, and the probability that a cyst is localized in a pork portion, and the probability that the cyst remains viable when undercooked.

The probability of exposure to TSTC was obtained as a product of the stated probabilities above.

### Statistical analysis

The stochastic Monte Carlo simulation with the aid of @risk software version 8.1 an add-on in Microsoft Excel was used for risk assessment in exposure assessment, hazard characterization, risk characterization, and sensitivity analysis. Furthermore, descriptive statistical analysis was done using IBM SPSS statistics version 21 to obtain the consumption pattern and preparation preferences of pork from the questionnaire survey.

## Results

### Demographic information

One hundred and fifty respondents were included in this study for a questionnaire survey. Of these, 88 (58.7%) were male, while 62 (41.3%) were female. Furthermore, 8% of the respondents did not attend any formal education, 63% attended primary education, 22% attended secondary education, and 6% attended college/university. Out of 150 respondents, 51.3% were farmers, 38% were self-employed in different sectors, and 4% were government employees (Table-2).

**Table-2:** The demographic information of respondents for pork consumption patterns and preparation preferences in the Mpwapwa district.

Gender			

Male			Female
88 (58.7%)			62 (41.3%)

**Level of education**			

**None**	**Primary school**	**Secondary school**	**College/University**

12 (8%)	95 (63.3%)	34 (22.7%)	9 (6%)

**Primary occupation**			

**Farmer**	**Government employee**	**Self- employed**	**None**

77 (51.3%)	6 (4%)	58 (38.7%)	9 (6%)

### Pork consumption and preparation preferences

#### Pork consumption pattern

The frequency of eating pork and the quantity of pork consumed in this study area varied from person to person with the main determinant being the availability of money for buying pork. Most people, about 98% reported buying an average of 500 g of pork meat any time when they get money, while only 2% reported buying pork only when there is a special local gathering. The monthly consumption showed that 34% of pork consumers ate pork thrice a month, while 24% consumed pork once per month (Table-3). About 19.3% and 43.3% indicated buying and consuming pork at local breweries and local pork butchers, respectively. Only 37.3% indicated preparing and consuming their pork in their respective homes (Table-4). On all occasions, the meal was shared with an average of three people.

**Table-3:** The frequency of pork consumption per month by different pork consumers in Mpwapwa district.

Consumption	Frequency	Percent	Valid percent	Cumulative percent
Valid				
Once	36	24.0	24.0	24.0
Twice	30	20.0	20.0	44.0
Thrice	51	34.0	34.0	78.0
Every week	9	6.0	6.0	84.0
Often	24	16.0	16.0	100.0
Total	150	100.0	100.0	

**Table-4:** The sources and places where pork is consumed by pork consumers in Mpwapwa district.

Source	Frequency	Percent	Valid percent	Cumulative percent
Valid				
At club/local brews	29	19.3	19.3	19.3
Butchery	65	43.3	43.3	62.7
Home	56	37.3	37.3	100.0
Total	150	100.0	100.0	

#### Pork preparation preferences

Two pork preparation methods were common in the area. These were boiling followed by deep-frying in hot oil and the other one was deep-frying in hot oil alone. Of the two methods, boiling followed by deep-frying in hot oil were the most preferred preparation as reported by 60% of pork consumers while deep-frying alone was preferred by 40% of pork consumers. However, most consumers who preferred boiling followed by deep-frying were from villages in the town centers of Mpwapwa town and Kibakwe Wards while those from rural villages preferred deep-frying alone (Table-5). On the other hand, 60% of pork consumers preferred complete dried pork while 40% preferred partially done pork. Furthermore, 10% of pork consumers used <10 min to complete their preferred preparation. In addition, 16 (10.67%) pork consumers in this study area expressed their preference for undercooked or raw pork. Among these, four pork consumers preferred raw pork and 12 people preferred undercooked pork. This included those who preferred partially done pork, deep-fried alone below 15 min.

**Table-5:** Pork consumer’s preparation preferences in Mpwapwa district.

Preparation preferences	Ward				Total (%)

	Kibakwe	Matomondo	Mpwapwa town	Pwaga	
Which is your preferable preparation					
Boiled and then deep-fried	26	7	48	9	90 (60)
Fried in oil alone	12	31	5	12	60 (40)
Total	38	38	53	21	150

#### Risk assessment

Results of the risk assessment are shown in Tables-6a and b [7, 8, 17, 20–22, 24, 27, 40–59].

**Table-6a:** The model parameter description numbers (n1–n9) and Probabilities (P1–P6) for the risk of getting exposed to *Taenia solium* taeniasis/cysticercosis through consumption of pork in the Mpwapwa.

Parameters	Input parameters description	Source of information	Probability distribution	Level of confidence (95% CI)
n1	Number of pigs slaughtered in Mpwapwa district per year	The meat inspection report from Mpwapwa district veterinary office	RiskIntUniform (5935, 7928)	6932.00
P1	The probability that the pig is infected at the farm	[7, 8, 41–43]	RiskPert (0.045, 0.17, 0.333)	0.26800
n2	Number of infected animals sent for slaughter	Product of n1 and p1(n1*p1)	RiskBinomial (6932, 0.176333333)	1222
P2	The probability that an infected pig is missed by lingual inspection	[22, 44–47]	RiskPert (0.306, 0.33, 0.92307)	0.60714
n3	Number of infected pigs not detected by lingual palpation	Product of n2*P2 in this study	RiskBinomial (1222, 0.398845)	487
P3	The probability that infected pigs are not detected at post-mortem inspection	[17, 21, 22, 45, 48]	RiskPert (0.49854, 0.774, 0.8)	0.732323
n4	Number of infected pigs not detected at post-mortem meat inspection	Is a product of P3*n3 in this study	RiskBinomial (487, 0.732323)	357.000
P4	The probability that infected pork contains viable cysts	[22, 47, 49, 50]	RiskPert (0.11, 0.7742, 0.909)	0.86983
n5	Number of infected carcasses containing viable cysts	Was a product of n4*P4 in this study	RiskBinomial (357, 0.68597)	259.000
n6	Meal potions per pig/person	[51–53]	RiskPert (262, 342, 400)	379.45
n7	Infective meal portions passed for consumption/year	Product of n6*n5 in this study	RiskPoisson (82,892)	83366.00
n8	Number of cysts per carcass	[54]	RiskIntUniform (2, 38,730)	19366.00
P5	The probability that cysts in an infected carcass are viable	[24, 55]	RiskPert (0.322, 0.7994, 0.89565)	0.86769
n9	Number of viable cysts in a carcass	Was obtained as a product of P5*n8	RiskBinomial (19,366, 0.7994)	15573.00
P6	The probability that a cyst is localized itself in a pork portion	[20, 56]	RiskUniform (0.106, 0.32)	0.99590

**Table-6b:** The model parameter description (number (n10–n22) and probabilities (P7–P11) for the risk of getting exposed to *T. solium* taeniasis/cysticercosis through consumption of pork in the Mpwapwa.

Parameters	Input parameters description	Source of information	Probability distribution	Level of confidence (95% CI)
n10	The number of cysts distributed in the meal pork portions	Was obtained as the product of (n9*P6) in this study	RiskBinomial (15481, 0.25)	3959.00
n11	Number of viable cysts per pork meal portion size destined for consumption	Was the ratio between the number of cysts distributed in the meal pork portions and the number of meals per pig (n10/n6)	RiskPoisson (5)	9,000
P7	The probability that cysts remain viable when undercooked	[57]	RiskUniform (0.033, 0.983)	0.95000
N	Population of Mpwapwa district	[27]	RiskPoisson (305,056)	305964
n12	Number of households in Mpwapwa districts	[27]	RiskPoisson (66,316)	66739.00
n13	The number of households consuming pork in Mpwapwa district	4.1% of households in Mpwapwa consume pork [40]	RiskBinomia (66,363, 0.41)	27417.00
n14	Number of people consuming pork in Mpwapwa district	Was obtained as a product of n2*3. Three was the number of people who shared this meal in this study	RiskPoisson (8157)	8306.00
P8	The probability of getting exposed to *T. solium* taeniasis	From this study	RiskUniform (0.018, 0.04633)	0.044912
n15	Number of people getting exposed *T. solium* through consumption of pork per year	Product of P8*n14 in this study	RiskBinomial (8157, 0.018767)	153
P9	A proportion of people who consume undercooked pork/per year	12/150 people expressed preferred undercooked pork in this study and [58]	RiskPert (0.08, 0.215, 0.35)	0.29887
n16	Number of people who consume undercooked/year.	Product of P9*n14 in this study.	RiskBinomial (8157, 0.215)	1815.00
n17	The number of people exposed by eating undercooked pork.	Product of P8*n14 in this study	RiskBinomial (1754, 0.032165)	69.000
P10	Probability of developing *T. solium* taeniasis infection.	From this study	RiskUniform (0.6882, 0.99951)	0.98392
n18	The number of people getting infected through consumption of undercooked pork.	Product of P10*n17 in this study	RiskBinomial (1863, 0.023333173	47.00000
P11	A proportion of people consume raw pork/per year.	4/150 expressed a preference for raw pork in this study and [59]	RiskUniform (0.02667, 0.21)	0.20082
n20	Number of people who consume raw/year	Product of P11*n14 in this study	RiskBinomial (8157, 0.12)	1027.00
n21	Number of people getting exposed by eating raw pork.	Product of n20*p8 in this study	RiskBinomial (979, 0.032165)	41.000
n22	Number of people getting infected through consumption of raw pork	Product of n21*P10 in this study	RiskBinomial (1754, 0.02667)	47

*T. solium*=*Taenia solium*

### Exposure assessment

The number of pork meal portions containing viable cysts at consumption in Table-6 was simulated at 100,000 iterations. It was found that 258, 542 (95% CI = 24227–260831) infective pork meal portions were released for consumption per year with a portion prevalence of 4.78%.

Routine meat inspection accounted for the removal of 756 (95% CI = 126–1061) infected carcasses equal to 61.87%, thereby releasing 466 (38.13%) infected carcasses into the food chain.

**Figure-6:**
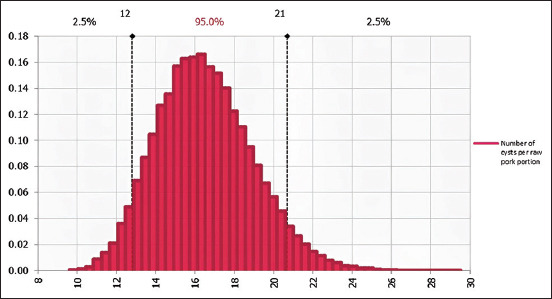
The number of viable cysts in 100gs raw pork portion at consumption.

This model predicted the number of viable cysts in a 100 g pork portion at consumption to be 17 cysts (95% CI = 12–21 cysts) in the raw pork portion (Figure-6) and eight viable cysts (95% CI = 0–18 cysts) in undercooked pork.

### Hazard characterization

#### Dose-response relationship

**Figure-7:**
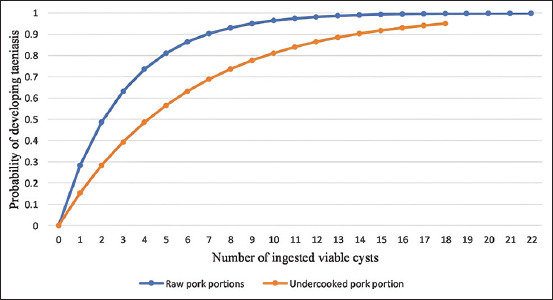
Combined dose-response relationship curve of raw pork and undercooked pork.

The dose-response relationship for raw and undercooked pork portion are plotted in Figure-7. From these dose-response graphs in this study, the probability of developing infection was found to be 0.99652 (95% CI = 0.9816–0.99908) when a 100 g raw pork meal is consumed and 0.73605 (95% CI = 0–0.95) from consuming a 100 g undercooked pork meal portion.

### Risk characterization

This model predicted that the risk of one developing TSTC through the consumption of pork approved for human consumption to be 0.018 (95% CI = 0.00–0.0250) The probability of developing *T. solium* taeniasis was estimated to be 0.73605 (95% CI = 0–0.950) when an infected undercooked pork portion is consumed compared to 0.99652 (95% CI = 0.98161–0.99908) from a raw pork portion.

**Figure-8:**
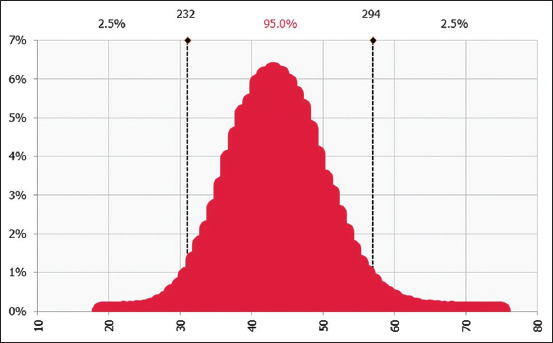
Number of people getting exposed to T. solium taeniasis through consumption of approved pork in Mpwapwa district per year.

**Figure-9:**
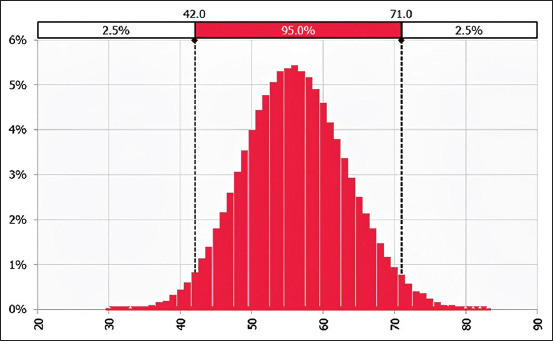
Number of people getting exposed to *T. solium* taeniasis through consumption of undercooked pork in Mpwapwa district per year.

**Figure-10:**
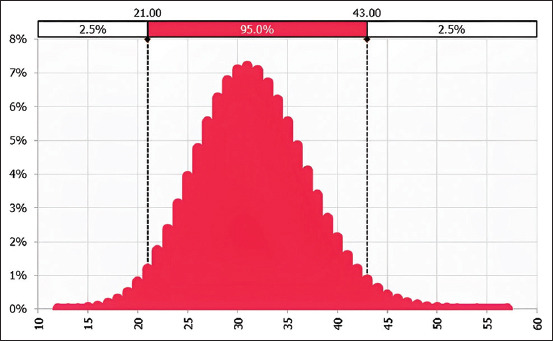
Number of people getting exposed to *T. solium* taeniasis through consumption of raw pork in Mpwapwa district per year.

Another Monte Carlo simulation set at 56,000 iterations was done to model the number of people getting exposed to TSTC per year. It was revealed that among 8157 possible pork consumers in Mpwapwa District, 262 (95% CI = 232–294) get exposed to the parasite through the consumption of pork per year (Figure-8). Moreover, 56 (95% CI = 42–71) of 1754 people who consume undercooked pork get exposed through the consumption of undercooked pork (Figure-9), while 31 (95% CI = 21–43) of 979 get exposed to the parasite through the consumption of raw pork per year (Figure-10). In addition, about 47 (95% CI = 42–52) people who consume undercooked pork are likely to get infected and develop clinical signs of TSTC through the consumption of undercooked pork, while 26 (95% CI = 22–30) people are likely to get infected through the consumption of raw pork in Mpwapwa District per year.

### Variability and uncertainties

The number of viable cysts in the pork portion varied from 0 in properly cooked pork to 22 in raw pork. The dose-response relationship varied with the status of ingested pork meal. The probability of developing taeniasis according to the dose-response relationship was 0.15338 and 0.28332 when a single viable cyst is ingested in undercooked pork and raw pork, respectively. Furthermore, risk analysis studies are associated with uncertainties which may provide unreliable results if not dealt with. In this study, uncertainties were taken away by subjecting each input parameter to a specific probability distribution function in @risk software. Likewise, information gaps on pork consumption and preparation preferences as significant input parameters in this study were filled by undertaking a questionnaire survey other than depending on the logical assumptions and information from elsewhere. These measures have increased the validity and reliability of this study. However, the paucity of published information on the prevalence of PCC could have affected these results. However, this was taken into consideration using the published studies on the prevalence of PCC from the neighboring district which was once part of Mpwapwa District.

### Sensitivity analysis to determine the risk factors

#### The Spearman rank correlation coefficient Tanardo graph

**Figure-11:**
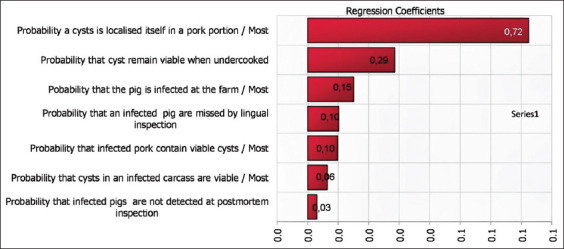
A most influencing risk factor to the exposure of infected pork meal as ranked by Spearman Rank Correlation Coefficient

The probability that a cyst is localized itself in a pork portion was the most influencing input to the risk of getting exposed to TSTC as it had a strong positive association of (ρ = 0.72) followed by the probability that the cyst remains viable when undercooked (ρ = 0.29) (Figure-11). Other inputs have the Spearman rank correlation coefficient ranking from 0.03 to 0.15, signifying weak association. However, this does not mean that they are not risk factors for exposure as the Spearman rank correlation coefficient does not measure the causal association but the relationship (association) between the variable input and output.

#### Change in output statistics Tornado graph

These graphs provide the most influencing input factor by ranking the inputs based on their effects on the mean of output. These graphs produced results similar to those from the Spearman rank correlation coefficient graphs whereby the probability that a cyst is localized itself in a pork portion was ranked as the top influencing input to the risk of getting exposed to TSTC (Table-7).

**Table-7:** The most influencing risk factor for exposure to *Taenia solium* taeniasis/cysticercosis as ranked by change in output statistic Tornado graphs.

Change in Output			

Rank	Name	Lower	Upper
1	The probability that a cyst is localized in a pork portion	1.51405E-12	0.013713
2	The probability that cysts remain viable when undercooked	0.000362	0.006527
3	The probability that the pig is infected at the farm	0.001727	0.005255
4	The probability that infected pork contains viable cysts	0.002106	0.004510
5	The probability that an infected pig is missed by lingual inspection	0.002465	0.004823
6	The probability that cysts in an infected carcass are viable	0.002588	0.004015
7	The probability that infected pigs are not detected at post-mortem inspection	0.003082	0.003739

## Discussion

This study aimed to quantify the risk of humans getting exposed to *T. solium* taeniasis through the consumption of pork approved for human consumption by employing a QRA model. This model was divided into two control points as applied in the pork processing chain. Each control point is significant in removing the infected pork from the food chain. These control points are the current routine inspection protocols (antemortem inspection at the farm or the slaughter premises and post-mortem inspection after slaughter) that the second control point is cooking and the control limits are the current specificity and sensitivity of meat inspection protocols, temperatures, and the time used to complete preparation of pork at cooking.

### Pork preparation preferences and consumption pattern

Results from the questionnaire survey on pork preparation and consumption pattern have shown that 60% of pork consumers in Mpwapwa District prepare their pork by frying in hot oil alone and 40% deep fry their pork after boiling it. Deep-frying in hot oil alone predisposes consumers to the risk of ingesting viable cysts as this method may not kill all cysts localized in the deep part of the pork portion [60]. The inefficiency of this pork preparation method in rendering pork safe for consumption is also reported by Møller *et al*. [57], stating that “deep-fried pork in oil may entail a risk due to generally shorter cooking time.” Deep-frying alone in hot oil as the method of pork preparation was highly preferred by respondents from rural villages in this study. People in urban areas are easily accessible to health education messages. Moreover, these residents have better health-seeking behaviors and higher levels of literacy than rural residents [61]. This then could be the attributing factor to the observed difference in pork preparation preference between people in rural and urban areas. This is because about 55.88% of the respondents with secondary education and 66.67% of the respondents with college/university education belong to only two villages that are in the town centers of Mpwapwa Ward and one village of Kibakwe Ward among eight villages in this study. Although, testing the statistical association between the level of education and pork preparation preferences was not done in this study.

Likewise, the saving of pork from local brew bars and clubs has been reported as the predisposing factor to the consumption of insufficiently cooked pork [62] from the southern highlands and [63] the northern zone of Tanzania. This is also witnessed in this study whereby about 29% of pork consumers in Mpwapwa District save pork from local brews bars and clubs. Heavy drinking of alcohol, lack of close supervision during cooking, and unhygienic condition in these areas are the culminating factors for the ingestion of viable cysts in pork [4, 23].

Furthermore, the consumption of undercooked or raw pork is a major risk factor for direct exposure to *T. solium* taeniasis and indirect exposure to *T. solium* cysticercosis [8, 43, 64]. In this study, about 10.6% of pork consumers reported preferring undercooked or raw pork; surprisingly this number of people who consume such food could be higher in this study area since consumers who save their pork meals at local butcheries commonly located near bars and local community gatherings, and those who buy ready to eat pork from the pork street vendor have low ability to decide on the pork preparation preferences at some points. This was observed during the researcher-respondent conversation administering the questionnaire, whereby one respondent quoted “*I prefer to eat well-cooked pork but pork seller have few frying pans*.” Another respondent reported that street pork vendors practice partial cooking of pork portion to attract customers; likewise, well-cooked pork portion decreases in size and hence becomes rejected “*people like big pork portions but when completely cooked they shrink and become rejected*.” Another pork vendor reported cooking for a short time when there is a long queue of people waiting. These results are consistent with results from other studies, which suggest that poor customer supervision and long queues are predisposing factors to eating undercooked pork among people who save pork from local brews bars [62].

### Quantitative risk assessment

This study revealed that pork consumers in Mpwapwa District are exposed to up to 22 viable cysts when consuming a 100 g pork portion size unlike what was reported by Borkataki *et al*. [65], who reported 89.5 ± 6.26 cysts per 50 g pork portion size in India. This high infection load in this study was because all ten pigs from which cysts were sampled were heavily infected with more than 100 cysts/carcasses. For this study, the level of infestation was taken into consideration during exposure assessment. However, these exposures in this study varied with whether the pork portions were being eaten raw or undercooked. Exposure from the raw pork portion and the probability of developing infection was 2 times higher compared to the undercooked portion. This was attributed to the lethal effect of heat on the viability, the infectivity, and the dose of ingested cysts in undercooked pork portions. More doses are ingested from raw pork portions compared to undercooked pork as some cysts are killed when the pork is subjected to cooking. This is revealed from this study whereby the number of viable cysts ranged from 0 to 18/100 g undercooked pork portions, while in raw pork portion, the number ranged from 12 to 22 viable cysts. Furthermore, this study argues that the virulence factors of viable cysts from undercooked pork are considered to be reduced and hence more susceptible to the host defense mechanisms, thereby failing to establish the infection. These arguments are similar to those arguments by Wakelin [37] and Paull *et al*. [66], who reported that high temperature causes thermal stress to pathogenic microorganisms and become more prone to destructive enzymes, immune responses, and other protective mechanisms of the host. This then reduces the efficacy of the cysts in establishing infection. Due to this, the likelihood of 0.283 and 0.1534 of a cyst to establish *T. solium* taeniasis infection in the human host when a raw and undercooked pork portion is consumed, respectively, was low in this study compared to 0.6 in Ito *et al*. [34] and Yoshino [36], where the viable cysts were just swallowed.

The study revealed further that the probability of getting exposed to TSTC through the consumption of pork approved safe for human consumption in Mpwapwa District is 0.018 (95% CI = 0.00–0.0250). These results are more or less similar to the results obtained in Kenya [67] which revealed the risk of getting exposed to viable cysts in pork portion at consumption to be 0.006 (99% CI = 0.0002–0.0167). These similarities could be attributed to similar geographical, epidemiological, risk factors, and endemicity of the disease. *Solium* taeniasis/cysticercosis is endemic in both countries [7, 23, 58, 68–70] compared to Europe [20], where the disease is not endemic and the probability of releasing infected pork to the food chain is low (0.0036%). The slight difference observed could be attributed to the level of uncertainty; this study included the probability that cysts remain viable when undercooked and the probability that a cyst localizes itself in the pork portion in modeling this risk of exposure. Likewise, the reported study in Kenya used much of the primary data directly from the field hence a high confidence level.

Furthermore, this study revealed that about 73 people get infected with *T. solium* taeniasis through consuming undercooked and raw pork per year and about 262 pork consumers (95% CI = 232–294) get exposed to the disease per year. These infection rates in this study are the factor of the prevalence of the consumption of raw and or insufficiently cooked, which in turn has been linked to the spread of *T. solium* taeniasis in many endemic areas [43, 64, 71]. The severity of these infections cannot be perceived only from the number of people being infected and the clinical manifestation of *T. solium* taeniasis in this study but also from the sequel of these infections. *Taenia solium* taeniasis infected persons act as the source of infection to PCC in pigs and NCC in humans [60] in case, where there is open defecation and when pigs access human feces from an infected person. Based on the reproduction number (*R_0_*) of *T. solium* taeniasis of 1.4 [72], 73 infected people were responsible for spreading *T. solium* cysticercosis to 73 new people in Mpwapwa District. Even if there are no studies on the prevalence of the disease in the district, three studies in the neighboring District which once formed part of Mpwapwa District reported the presence of *T. solium* eggs in soil [73] and the prevalence of PCC in pigs [42, 74] indicating that this disease might have been be endemic in Mpwapwa District.

With regard to the sensitivity of meat inspection, this was modeled in the present study based on portion prevalence. Portion prevalence provides the ratio of the number of infective meal portions released into the food chain to the total infected pork meal detected at the official meat inspection. Therefore, portion prevalence is a factor of sensitivity and specificity of both antemortem and post-mortem inspections. In this study, the meat inspection protocol was responsible for removing only 61.87% of infected carcasses, thereby releasing 38.13% of infected carcasses, which is equivalent to 108, 266.667 infective pork meal portions, into the food chain. The study by Sciutto *et al*. [49] elsewhere provides similar sensitivity to official meat inspection, where 60%–64% of infected pigs were detected by post-mortem meat inspection which involved tongue dissection. Other studies also attributed the limitations of official meat inspection to its low sensitivity and hence tended to miss light infected pork, although it (official meat inspection) performs better for heavily infected pork [47]. The study by Dorny *et al*. [45] gives the sensitivity of antemortem inspection to be 0.210 and of post-mortem inspection to be 0.211. Phiri *et al*. [22] report the sensitivity of antemortem and post-mortem inspection to be 16.1 and 38.87 official meat inspection, respectively, while Meester *et al*. [20] report the sensitivity of meat inspection protocol to be 0.32. All these studies communicate a similar message, that is, even if the current meat inspection protocol is simple to perform and cheap, t it is not able to remove all infected pork in the food chain. Similarly, Thomas *et al*. [48] report the prevalence of PCC to be 37.6% (95CI 19%–49.9%) among pork released for human consumption, while no infected carcass was detected at post-mortem meat inspection.

### Sensitivity analysis of the risk factors for exposure to TSTC

Sensitivity analysis was done to identify the most significant input parameter that contributes most to the probability of exposure. Both the Spearman rank correlation coefficient and the change in output statistics Tornado graph indicated that the probability that a cyst localized itself in a pork portion is the most influencing input to the risk of getting exposed to TSTC, having a strong association of (ρ = 0.72) followed by the probability that cyst remains viable when undercooked, having a week association of (ρ = 0.29). The results from this analysis show that one cannot get *T. solium* taeniasis infection unless a pork portion containing a viable cyst is ingested. When the pork is undercooked, the fat content of pork portions insulates the cysts; likewise, those cysts located in the deep part may withstand that cooking temperature [57, 75, 76]. These arguments agree with the arguments in studies elsewhere that report a high prevalence of *T. solium* taeniasis being associated with the consumption of infected raw or undercooked pork [43, 77, 78]. For this reason, results from this analysis indicate that pork consumers are the only assurers of pork safety as they are at the end of the pork chain and meat inspection cannot remove all infected carcasses from the food chain.

## Conclusion

This study has revealed that pork consumers in Mpwapwa District are at risk of being exposed to TSTC through the consumption of routinely inspected pork. Therefore, there is a need to establish an official meat inspection protocol that will have a significant sensitivity and which will still be affordable and doable as opposed to that of the golden standard of full carcass dissection. Likewise, it is high time to have joint efforts for stimulating the interest of policymakers toward resource allocation as well as employing a one-health approach for enhanced and improved sanitation and pig husbandry practices in Mpwapwa District and the country at large. In addition, this study suggests sensitization to pork consumers and the whole population, especially in rural areas on the proper method of pork preparation.

## Authors’ Contributions

MAM, KEM, and EM: Contributed to conceptualization, study design, and data analysis. MAM and EM: Prepared the model input parameters. MAM: Contributed to data curation and drafted the manuscript. MAM, KEM, and EMM: Contributed to the editing of the final draft, supervision, and validation. All authors have read, reviewed, and approved the final manuscript.
